# 1163. HIV-1 NAAT Pitfalls in B-cell Lymphoblastic Leukemia Patients Following CAR-T Cell Therapy

**DOI:** 10.1093/ofid/ofab466.1356

**Published:** 2021-12-04

**Authors:** Muayad Alali, John Christenson, Jodi skiles

**Affiliations:** 1 Indiana university, Carmel, Indiana; 2 Indiana University, Indianapolis, Indiana; 3 indiana university, Indianapolis, Indiana

## Abstract

**Background:**

CAR-T Cell Therapy is approved for the treatment of pediatric patients with relapsed/refractory B-ALL. Lentiviral vector technology, highly modified from HIV-1, is used to induce stable, long-term transgene expression by integration into the host genome. This integration may interfere with HIV-1 NAAT producing false-positive results.

**Methods:**

A retrospective chart review of Pre-B ALL patients who underwent to CAR T cell therapy with KYMRIAH(tisagenlecleucel) at a single institution between January 2019 and May 2021 to assess for patients whose CAR-T infusion interfered with post-infusion HIV-1 testing. All patients had HIV NAAT pre and post CAR T-cell therapy (using platform, Roche COBAS AmpliPrep/Quantitative TaqMan HIV-1 T). Reactive HIV NAAT by Roche test were subsequently tested using different HIV-1 assay platforms to rule out or confirm HIV infection.

**Results:**

We report three cases in which interpretation of HIV-1 NAAT testing was complicated by CAR T cell therapy. Case 1: 1-year-old male with refractory infantile leukemia was found to have a reactive HIV NAAT post CAR-T in routine infectious screening prior to SCT. Case 2: 3-year-old refractory ALL planned for SCT who had a reactive HIV NAAT 9 months post CART. Fourth-generation HIV-1 testing (targeting the p24 antigen and anti-HIV-1 antibodies) was negative pre and post CART. Viral loads were also undetectable indicating false-positive post-CAR-T HIV-1 NAT test results. Case 3: 21-year-old sexually active female with relapsed B cell ALL. Post CAR-T HIV NAAT was reactive, and a quantitative viral load was positive at 176 copies/mm^3^ one day prior to start of stem cell transplant conditioning regimen. Aptima HIV testing is typically used as confirmatory test for CAR-T associated false positive HIV NAT cases, but surprisingly, the Aptima test was also reactive. Additional testing with Abbott m2000 Realtime HIV-1 assay and 4th generation p24 antigen-based testing were both negative.

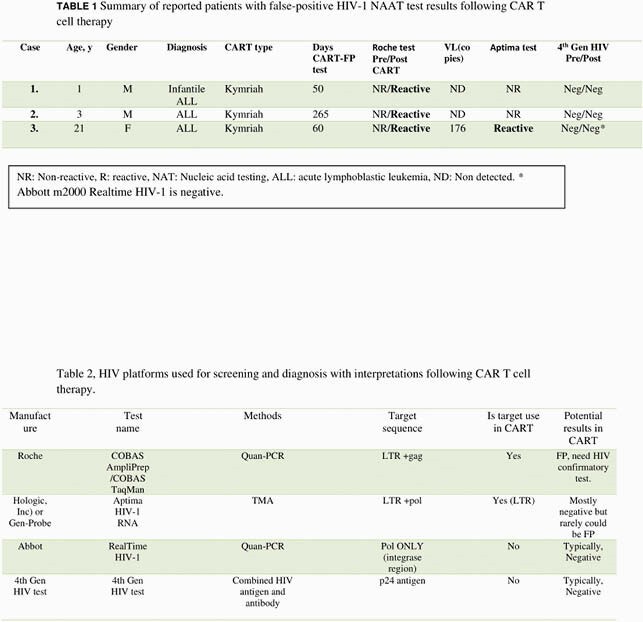

Table 1 Summary of reported patients with false-positive HIV-1 NAAT test results following CAR T cell therapy and Table 2, HIV platforms used for screening and diagnosis with interpretations following CAR T cell therapy.

**Conclusion:**

Clinicians need to be aware of potential false-positive HIV testing after CAR-T therapy. HIV testing platforms with targets not used in lentiviral vectors (4^th^ generation test/P24, or Abbott test/integrase region) are highly recommended to avoid delays in subsequent therapy and unwanted stress for the patients, families, and clinicians

**Disclosures:**

**All Authors**: No reported disclosures

